# The Role of Perceived Social Support on Quality of Life in People with Cardiovascular Diseases

**DOI:** 10.4314/ejhs.v32i5.17

**Published:** 2022-09

**Authors:** Khalil Maleki Chollou, Shayesteh Shirzadi, Sara Pourrazavi, Towhid Babazadeh, Soheila Ranjbaran

**Affiliations:** 1 Department of Nursing, Sarab Faculty of Medical Sciences, Sarab, Iran; 2 Shayestehshirzadi, PhD in Health Education and Promotion, Department of Public Health, School of Health, neyshaburunivercity of Medical Sciences, neyshabur, Iran; 3 Department of Health Education and promotion, Tabriz University of Medical Sciences, Tabriz, Iran; 4 PhD in Health Education and Promotion, Department of Public Health, Sarab Faculty of Medical Sciences, Sarab, Iran

**Keywords:** Social support, Quality of life, Cardiovascular Diseases, Iran

## Abstract

**Background:**

Cardiovascular Diseases (CVDs) are one of the major causes of hospitalization and mortality worldwide. Strengthening perceived social support and quality of life can reduce these problems. This study aimed to describe the role of perceived social support on cardiovascular patients' quality of life.

**Methods:**

This cross-sectional study was conducted from September 2020 to February 2021. We selected 150 cardiovascular patients through convenience sampling. The questionnaires included: the Persian version of the WHOQOL-BREF, Perceived Social Support, and demographic variables. Hierarchical linear regression was used to explore the association between perceived social support and quality of life. Data were analyzed by SPSS version 21.0 software. A P-value less than 0.05 is considered statistically significant.

**Results:**

The demographic variables could predict 12.2% of the variance of quality of life in the first step. In the second step, after adjusting control variables and dimensions of social support, the predictability increased to 29% of the variance with the addition of variables. All dimensions of social support, excluding tangible assets support, were significant predictors of quality of life and monthly income status. Self-esteem support (β= 0.387) was the strongest predictor of quality of life in cardiovascular patients.

**Conclusions:**

Patients with higher perceived social support have a better quality of life than the other patients. Perceived social support is one of the strategies that can be utilized to improve quality of life and overcome disease in cardiovascular patients.

## Introduction

According to the WHO's definition, “CVDs are a group of disorders of the heart and blood vessels and include coronary heart disease, cerebrovascular disease, rheumatic heart disease, and other conditions” (1). CVDs are thefirstcause of death worldwide, taking an estimated 18.6 million death each year (2). CVDs account for 45% of all deaths in Europe and 37% in the European Union (EU). Also, it is responsible for the loss of more than 64 million DALYs in Europe (23% of all DALYs lost) and 26 million DALYs in the EU (19%) (3). The greatest burden of CVDs is in low and middle-income countries (LMICs), with approximately >75% of cardiovascular deaths (4). Among the 18.6 million CVDs deaths worldwide in 2019, 58% occurred in Asia (5). In Iran, CVDs are the first leading cause of mortality, and a million disability-adjusted life years (DALYs) led to 46% of all deaths and 20%–23% of the burden of disease in Iran (6).

Current global health policy goals include a 25% reduction in premature mortality from non-communicable diseases by 2025 (7). While current strategiesfor managing patients with CVDs are designed toreduce morbidity and prolong survival, treatmentshouldalso be focused on improving patients' QOL by reducingtheir symptoms, optimizing life's daily functions, and overall wellbeing(8). Quality of life is an essential part of evaluating health status (9). The World Health Organization defines QOL as “a broad-ranging concept affected in a complex way by the person's physical health, psychological state, level of independence, social relationships and their relationship to salient featuresof their environment” (10). Cardiovascular disease has a negative effect on the quality of life in such patients and effect all aspects of patients' lives (11, 12). Based on the results of studies, coronary heart disease is significantly associated with impaired QoL (11, 13) and QOL is low in patients with CVDs (14–16). There is evidence that supports the variables such as depression (17), readmission (18), lower educational level, andbeing single (19), and multimorbidity (20) effect on QoL in CVDs.

One of the effects of the factors on the quality of life is social support (21). Social support refers to the various types of free assistance from a social network, which may be formal and informal, including emotional, instrumental, informational, and appraisal (22). Social support is one of the most reliable predictors of disease morbidity and mortality (23) that serves as a buffer to prevent or reduce the harmful longer-term health effects associated with encountering undesirable and traumatic events (24). The absence of social support is a significant risk factor for poor prognosis in cardiac patients, and some evidence supports their independence in predicting adverse outcomes (25). In examining 300,000 participants from around the globe, including theUnited States, Europe, Asia, and Australia, the results showed that overall social support was associated with a 50% increased likelihood of survival (OR = 1.50) (26). Given the importance of QoL and social support in cardiovascular patients, the current study aimed to explain the role of perceived social support on quality of life among patients with cardiovascular diseases in Iran.

## Methods

**Study design and participants**: This analytical cross-sectional study was conducted in 2020. We aimed to investigate associations between QoL and PSS in patients with cardiovascular diseases. The study population was Cardiovascular Patients in Emam Khomeini Hospital of Sarab, Iran. We selected 150 cardiovascular patients through census sampling. Inclusion criteria werethe physical and mental ability toparticipate in the study, the absence of aggravating cardiovascular disease, and agreement to participate. Exclusion criteria include disease exacerbation, death, lack of significant medical history, and voluntary withdrawal.

In the case of accepting the invitation, the participants were set an appointment in Emam Khomeinihospital. At the beginning of the appointments, the participants were informed about the aim of the study and assured of the confidentiality of data, and finally, all signed a consent form Informed. At the time of appointments, the trained interviewer referred to the hospital and collected data at the hospital, and those who were illiterate were assisted in completing the questionnaire. In addition, participants were free to withdraw or remain in the study during data collection. Data collection was conducted from September 2020 to February 2021.

**Measures**

**Socio-demographic and Health-related Variables**: The participants' socio-demographic characteristics, namely, age, gender, marital status (married and single/ divorced/ widowed), educational level attained (illiterate, elementary, secondary school, high school, associate &bachelor's degree, and master of degree and above), employment status (employed, unemployed andself-employment), place of residence (urban or rural), number of family members (<4, 5 to 10 and ≥ 10), the family history of the disease (yes, no) and monthly income level (lower than 200 dollars and 200 dollars and higher) were obtained.

**World Health Organization's Quality of Life Questionnaire (WHOQOL-BREF)**: The Persian version of the WHOQOL-BREF contains 26 items that were used to obtain the necessary data on the Quality of Life. The validity and reliability of this questionnaire were assessed by Nejat et al. (27). The instrument assessesthequality of life in four broad areas:physicalhealth (7 items; α= 0.70), psychological health (6 items; α= 0.73), social relationships (3 items; α= 0.55), and theenvironment (8 items; α= 0.84) with five-point Likert-type response options. The overall score value for an individualrespondent could range from 26–130, with higher scoresindicating a better quality of life.

**Perceived Social Support**: Perceived social support was assessed by the interpersonal support evaluation list (ISEL-SF) (28). Ranjbaran et al. documented Iran's scales' psychometric properties (29). These four subscales are (a) AppraisalSupport, the perceived availability of someone to discuss issues of personal importance, (b) Tangible AssetsSupport, the perceived availability of material aid; and (c) Belonging Support, the perceived availability of others to interact with socially, and (d) Self-Esteem Support, the perceived availability of others with whom one compares favorably. The instrument, a four-point Likert-type scaling (definitely true, probably true, probably false, and definitely false; scored 0–3), was adopted. The overall score value for an individual respondent could range from 0–48, withhigher scores indicating greater perceived social support. Cronbach's α for the four subscales wereappraisalsupport (0.71), tangible assets support (0.74), belonging support (0.81), and self-esteem support (0.78).

**Statistical analysis**: We performed all the analyses using SPSS 21 (SPSS Inc, Chicago, IL, USA) and presented the data by mean (SD) and frequency (percent) for quantitative and qualitative variables, respectively. Also, the Kolmogorov-Smirnov test was used to test the normality. We applied a hierarchical linear regression analysis in two steps. In the first step, Socio-demographic variables were entered into the analysis using the enter method, and in the second step, we entered the Socio-demographic variables with Health-related Variables in the analysis using hierarchical linear regression. We considered the significance level for entry variables in the hierarchical linear regression model as 0.05. We also used the Independent Samples T-Test, One-way analysis of covariance (ANCOVA), Pearson Correlation, and Hierarchical linear regression to assess the relationship between Sociodemographic, Health-related Variables, Quality of Life, and Perceived Social Support.

## Results

A total of 150 cardiovascular disease participants participated in the study. The majority of the study participants were men (51.3%) in their 50s and older (66.9%). According to the findings, there was a statistically significant association between occupational status, education level, monthly income status with quality of life. Furthermore, only occupational status and monthly status had a statistically significant association with overall social support among the demographic factors. Table 1 details demographic characteristics and their relationship with quality of life and total social support.

**Table 1:** Demographic characteristics of the study participant and their association with QOL and total social support

Variables		F (%)	Quality of life	p-value	Total social support	p-value
	
			Mean± SD		Mean± SD	
Age groups (years)	50 >	50 (33.7)	51.74±7.86	0.141	42.26±5.07	0.226*
	50 ≤	100(66.9)	49.55±8.85		43.33±5.08	
Gender	Male	77 (51.3)	50.59±8.62	0.643	43.49±5.36	0.199*
	Female	73 (48.7)	49.94±8.57		42.75±4.75	
Job	Employed	39	54.69±6.50	0.001	41.82±5.27	0.042**
	Unemployed	57	49.24±8.68		42.49±4.67	
	Housewife	54	48.18±8.76		44.31±5.15	
Marital status	Married	135	50.15±8.52	0.596	43.11±5.07	0.298*
	Single	15	51.40±9.24		41.66±5.20	
Level of education	Illiterate	45	48.95±9.31	0.006	43.06±4.72	0.214**
	Under diploma	71	49.15±7.97		43.53±5.39	
	Diploma and higher	34	54.38±7.70		41.67±4.79	
Income (monthly)	200 dollars	78	47.62±8.67	0.001	43.87±4.79	0.024*
	200 and higher	72	53.15±7.52		42.01±5.24	

* p-value based Independent T-test

** p-value based one-way ANOVA test

Table 2 shows the dimensions of social support and quality of life as determined by the Pearson correlation test. According to this test, assessment support (r= 0.176) and self-esteem support (0.327) had a significant positive correlation with quality of life. The strongest correlation between self-esteem support and quality of life was discovered.

**Table 2:** Bivariate correlation between social support dimensions, and QOL

Variables	1	2	3	4	5	Mean ± SD
1= Appraisal Support	1					11.40 ± 2.07
2= Tangible Assets Support	0.268*	1				10.01 ± 20.8
3= Belonging Support	0.259**	0.250*	1			11.16 ± 1.66
4= Self-Esteem Support	0.032	0.190*	0.383*	1		10.40 ± 2.03
5= QOL	0.176*	0.085	0.132	0.327*	1	50.28 ± 8.57

* Correlation is significant at the 0.05 level (two-tailed)

The most important factors and changes in quality of life were predicted using hierarchical linear regression analysis (Table 3). The variables could predict 12.2 percent changes in quality of life in the first step, which included all demographic variables. Monthly income status is one of the demographic characteristics that can predict quality of life. In the second step of hierarchical regression analysis, dimensions of social support were included in the model to predict quality of life and demographic variables. The predictability increased to 29% with the addition of variables. All dimensions of social support, excluding Tangible Assets Support, were significant predictors of quality of life and monthly income status. Self-esteem Support (β= 0.387) was the most important predictor of quality of life in cardiovascular patients among these variables.

**Table 3:** Hierarchical linear regression of Quality of life on demographic variables and social support

Step/variables	β** (Step 1)	p-value*	β** (Step 2)	p-value*
**(1)** Age groups	-0.026	0.778	-0.102	0.240
Gender	0.066	0.447	0.01	0.994
Job	0.147	0.213	0.086	0.441
Marital status	0.020	0.818	0.043	0.595
Level of education	0.013	0.917	0.044	0.693
Income (monthly)	0.251*	0.016	0.221	0.021*
**(2)**Appraisal Support			0.158	0.041*
Tangible Assets Support			0.042	0.596
Belonging Support			0.261	0.002*
Self-Esteem Support			0.387	0.001*
R^2^	0.122	0.004	0.290	0.001*

* p<0.05

** β is standardized coefficient

## Discussion

This study investigated the role of perceived social support on quality of life among Iranian patients with CVDs. Findings of regression modeling indicated that monthly income status and all dimensions of social support, excluding tangible assets support, were significant predictors of quality of life. Among social support dimensions, we found that self-esteem support is the most important predictor of quality of life in cardiovascular patients.

According to the literature, social support has a positive and significant association with the heart patients' quality of life and has better outcomes for them (30–33). Increasing social support among heart patients can reduce depression, hospitalization, and mortality and increase self-care behaviors, health problems management, and overall quality of life (34,35). In fact, heart diseases can lead to a wide range of physiological, psychological, socioeconomic, and familial problems (31,32). These problems can cause frustration and disappointment and negatively affect the patients' quality of life (31). Patients who receive adequate social support can better overcome these problems and better adapt psychologically to their disease (32). Helgeson (2003) believes that social support improves mood, encourages people to participate in social activities, increases health behaviors, and overall improves quality of life. People on a social network persuade each other toward healthy behaviors (36). High perceived social support leads to enjoyment of recreational activities, a better feeling of life, and life satisfaction (37).

In the present study, self-esteem support was identified as the strongest predictor of quality of life inpatients with CVDs. This result was consistent with Helgeson's (2003) and Friedman and King's (1994) studies (36, 38). Self-Esteem support, also known as emotional support or appraisal support, is the provision of empathy, love, affection, trust, acceptance, intimacy, encouragement, and caring from social support sources such as family and friends. Providing emotional support lets the patients know they are valuable to others (39). As a result, they feel valued, loved, and cared for and are better able to overcome illness outcomes and achieve psychological well-being (33).

Studies examining the relationship between heart patient demographic characteristics and quality of life have found that gender, age, education, marital status, employment status, duration of illness, and frequency of hospitalizations affect patients' quality of life. Among the various factors, our study found an association between occupational status, education level, income status with patients' quality of life. Heart disease changes a person's life as it increases dependence on others for daily and social activities and financial worries and reduces job opportunities (31). Unemployment has been associated with an increased CVDs burden (40). Low income, loss of job, or working days due to illness can create a stressful environment for patients and their families (41). On the other hand, low income reduces the patient's access to costly medical care or increases receiving lower-quality care, and therefore, these negatively influence health and quality of life (40,41).

In addition, according to the literature, there was an inverse relationship between educational level and heart disease. Educational levels may affect heart patients' health in several ways. Individuals with low education tend to have an increased number of CVDs risk factors such as smoking, obesity, physical inactivity, and hypertension. Researchers also found a strong correlation between education and health literacy as a potential contributor to CVDs risk. Individuals with poor health literacy are not more likely to be able to adhere to their medications properly and thus experience more problems that may be contributing to their health and quality of life (40).

The result of this study indicated an association between occupational and income status and social support. Individuals with an occupation have a larger social network and receive more social support (42). Given the economic situation in Iran, it seems that access to more income and having a stable job that guarantees the living expenses and treatment of the patient increases receiving social support from family, friends, and relatives.

We recognized the role of perceived social support on quality of life in patients with cardiovascular diseases. Monthly income status and dimensions of social support such as appraisal support, belonging support and self-esteem support were significant predictors of quality of life in these patients. Meanwhile, self-esteem support was the most important predictor of quality of life in cardiovascular patients among social support dimensions. Hence, designing educational programs based on individual factors without addressing social factors cannot improve quality of life in patients with cardiovascular diseases. In summary, the perceived social support seems to be an important external factor in CVDs management programs and may reduce the emotional burden of disease and health care costs.
